# Cryptogenic Epidural Cervicothoracic Abscess: A Case Report and Literature Review

**DOI:** 10.7759/cureus.52189

**Published:** 2024-01-12

**Authors:** Harvey Misael Aguilar Mora, Julio Cesar Soto Barraza

**Affiliations:** 1 Neurosurgery, Hospital Juárez de México, Mexico City, MEX

**Keywords:** transverse myelitis, subdural empyema, thoracic spinal, cervical spinal, spinal epidural abscess

## Abstract

A spinal epidural abscess (SEA) is a rare infection characterized by pus formation in the spinal epidural space, associated with various degrees of motor, sensory, or combined deficits. It is linked to several risk factors and predominantly impacts middle-aged men. This report discusses an atypical case of a patient without any predisposing factors who developed a cervicothoracic SEA associated with significant transverse myelitis. A targeted literature search was conducted on PubMed, Scopus, and SpringerLink, employing terms such as "spinal epidural abscess, subdural empyema, and transverse myelitis." While there are numerous studies on this topic with a multidisciplinary approach, reports of cryptogenic SEA associated with the extensive involvement of cervical and thoracic spinal segments are rare. SEA is a very uncommon condition. Hence, a comprehensive understanding of its clinical presentation is crucial for adopting an appropriate diagnostic approach and delivering timely treatment.

## Introduction

Spinal epidural abscess (SEA) is a rare diagnosis with an incidence of approximately 0.2-2 cases per 10,000 hospital admissions [1]. Risk factors for SEA include diabetes mellitus, trauma, intravenous catheters, drug use, and alcoholism [2]. It is uncommon for these abscesses to occur in patients without comorbidities. The condition predominantly affects males and occurs in the middle age. The most common infectious agent is Staphylococcus aureus (S. aureus) (50-90%), followed by Gram-negative bacilli (10-17%) and Streptococcus (8-17%), while 5-10% of SEA are polymicrobial in origin [3]. Hematogenous spread accounts for a significant proportion of SEA cases, and they are generally bacterial in etiology, with S. aureus being the most commonly cultured species [2]. Typically, at the time of diagnosis, the abscess spans multiple segments, with the majority situated in the posterior region. Abscesses found anteriorly are generally associated with vertebral osteomyelitis [4].

SEA, a nosological entity, commonly affects the thoracic spine and presents with fever and paraparesis. Symptoms typically evolve over hours to days [5]. However, the classic clinical triad of back pain, fever, and neurological deficit manifests in only a minority of patients [6]. The development of SEA can be categorized into four stages: stage I involves back and/or neck pain at the level of the affected vertebral column, along with fever; stage II is characterized by radicular pain radiating from the affected part of the spinal cord; stage III exhibits neurological deficits such as hypoesthesia, motor weakness, and bowel or bladder dysfunction; and stage IV involves progression to paralysis [5].

MRI is the gold standard for diagnosing myelitis and spinal cord abscesses. There is hypointensity in T1 and hyperintensity in T2 due to edema, while the infected area shows slightly less hyperintensity in T2 than the edema in a non-vascular distribution [7]. The preferred treatment approach typically involves prompt surgical debridement along with intravenous antibiotics [8].

## Case presentation

**Figure 1:**
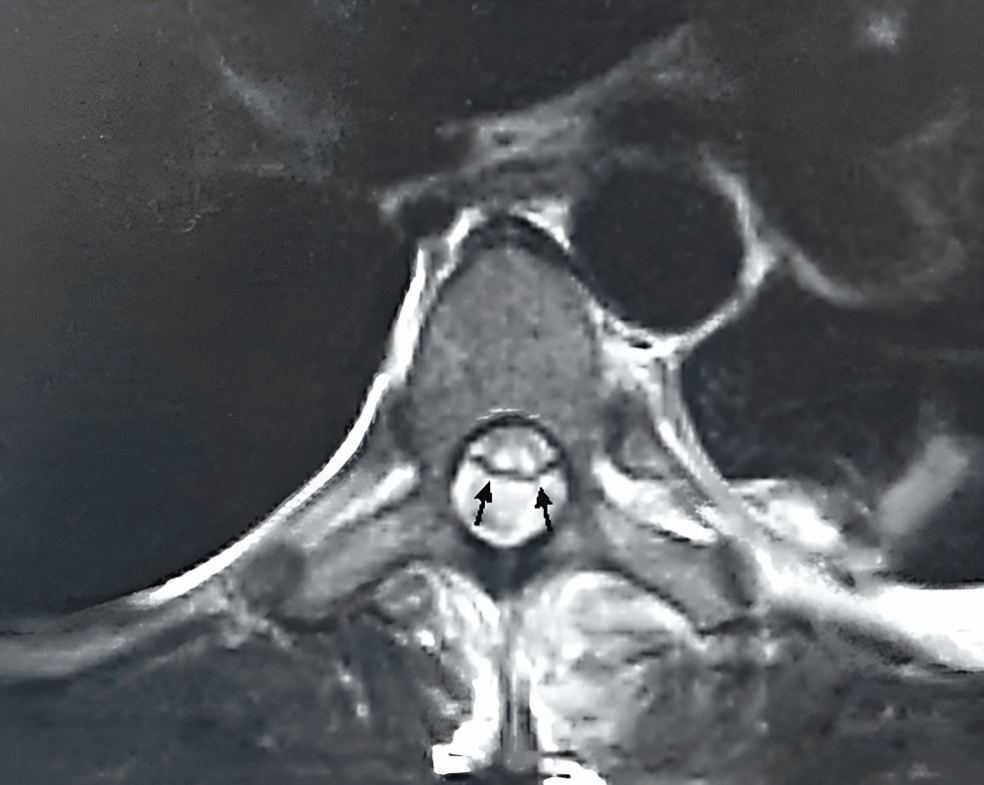
Axial T2-weighted MRI The image demonstrates an intradural-extradural spinal epidural abscess in the dorsal region at the T1 level, with ventral displacement of the spinal cord (arrows) MRI: magnetic resonance imaging

**Figure 2:**
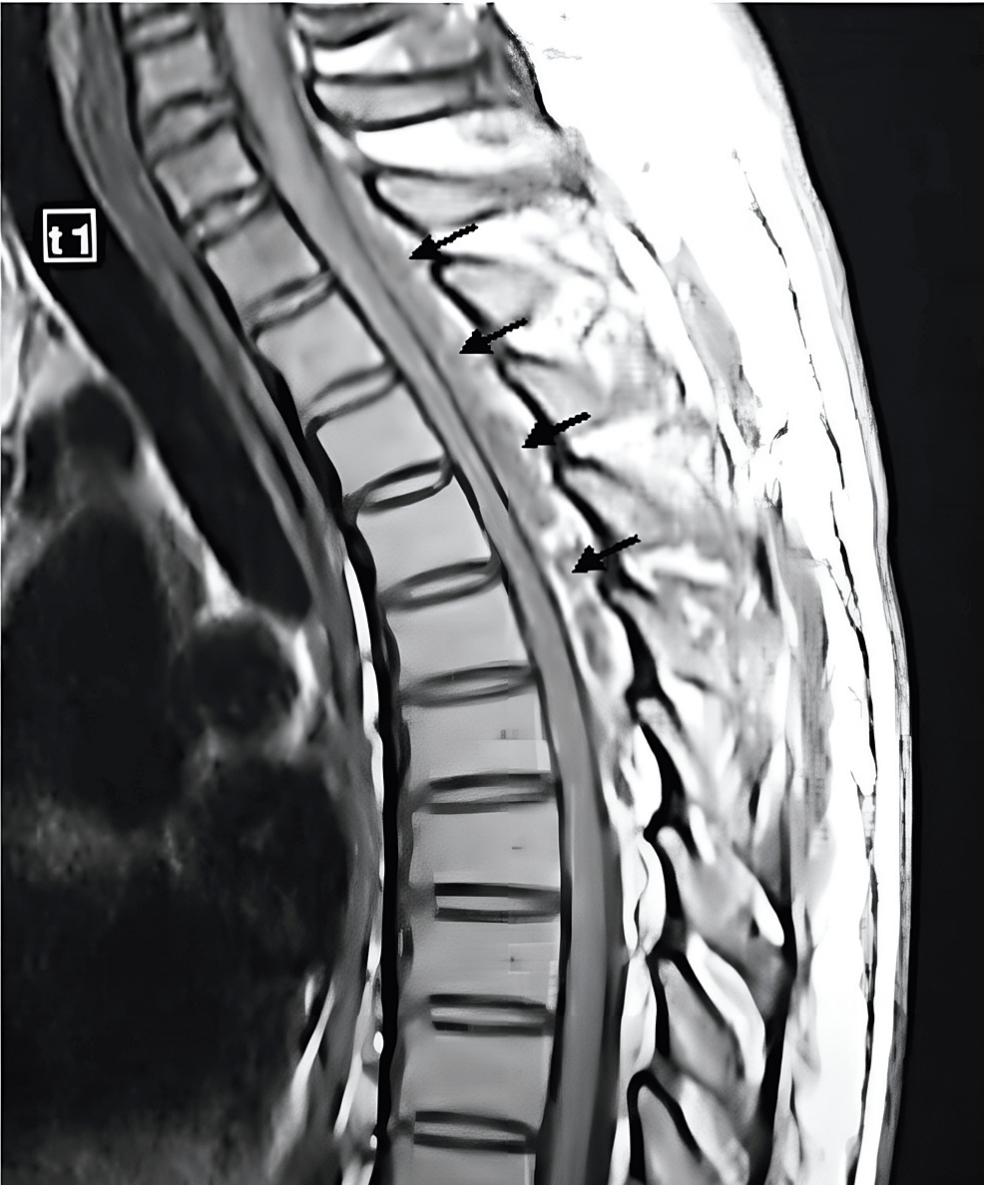
Sagittal T1-weighted MRI The image demonstrates a poorly defined and heterogeneous cervicothoracic spinal epidural abscess at C6-T10. There are hypointense and isointense images in the posterior region of the spinal canal MRI: magnetic resonance imaging

**Figure 3:**
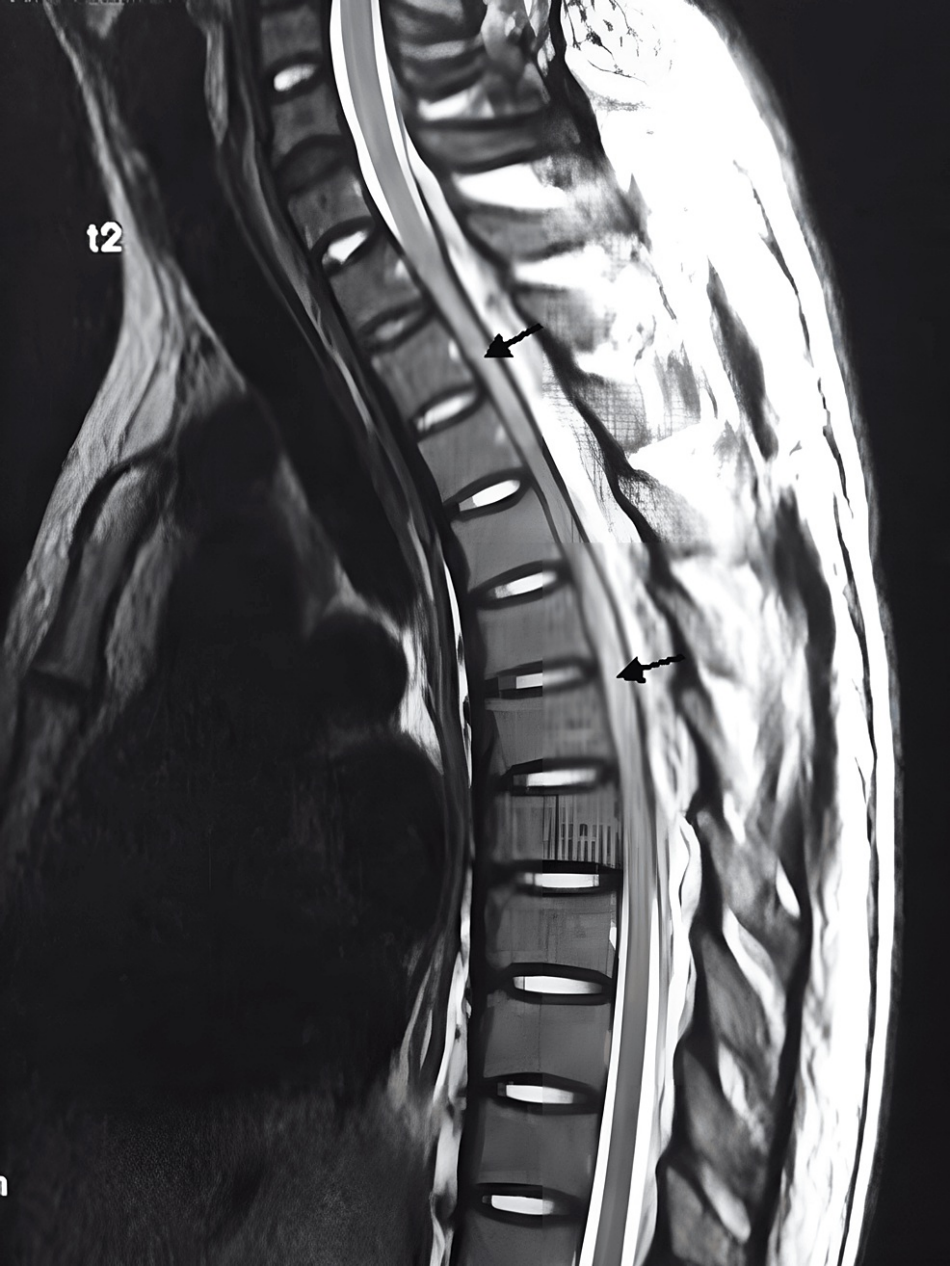
Sagittal T2-weighted MRI The image showing a cervicothoracic spinal epidural abscess at C6-T10 and hyperintense intramedullary images corresponding to areas of myelitis MRI: magnetic resonance imaging

**Figure 4:**
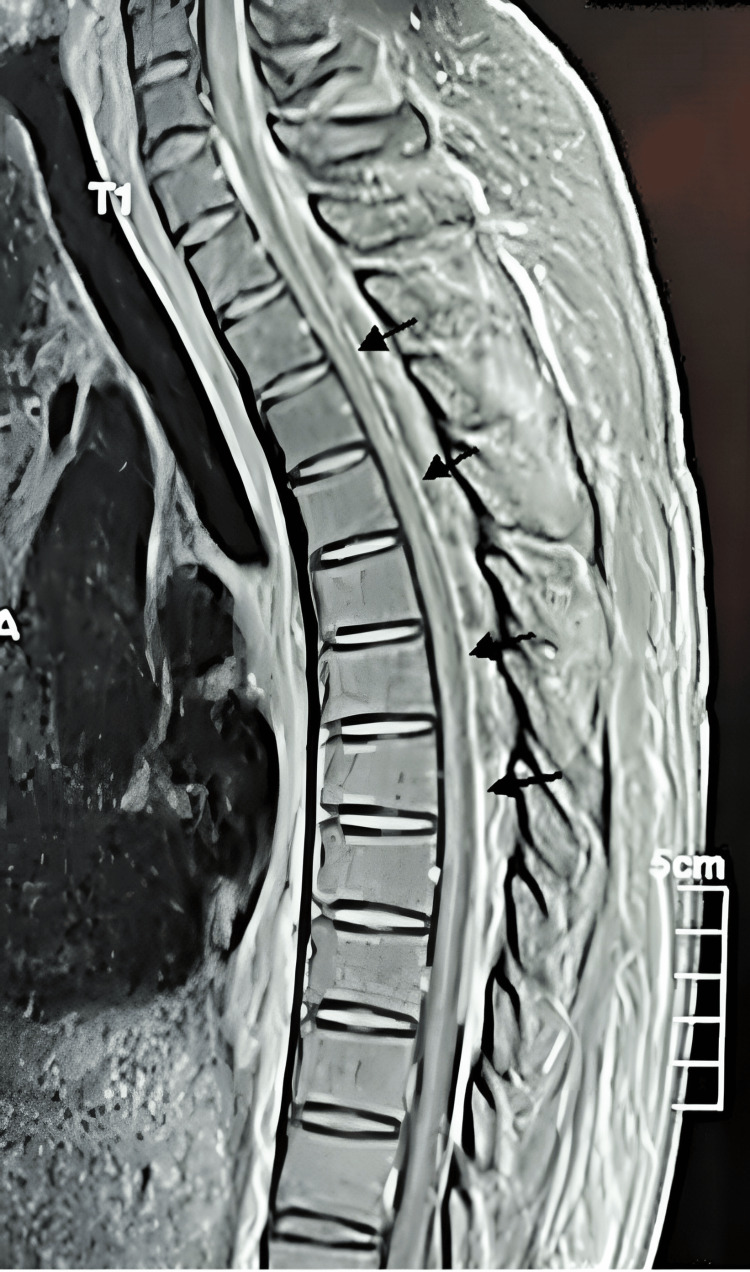
Sagittal T1-weighted MRI with gadolinium administration The image shows a heterogenous cervicothoracic spinal epidural abscess at C6-T10 with hypointense and isointense images in the posterior region of the spinal canal and hyperintense linear enhancement, predominantly in the ventral region (arrows) MRI: magnetic resonance imaging

A 19-year-old male, with no significant personal medical history or reported intravenous drug use, presented in good overall health. One week before the admission, he had complained of right-sided chest pain and an unspecified fever. Subsequently, he had developed symptoms such as constipation, abdominal distension, urinary retention, paresthesias, and muscle weakness in the lower extremities, resulting in impaired mobility. Upon admission to the emergency room, he was afebrile, with a heart rate of 78 beats per minute, a respiratory rate of 18 breaths per minute, and a blood pressure of 110/70 mmHg. Neurological examination revealed areflexic paraparesis and anesthesia from the T4 dermatome. The echocardiogram showed no abnormalities in mobility, thrombi, vegetations, or shunts, with a left ventricular ejection fraction (LVEF) of 55%. Electromyography of all four limbs, including F-waves and somatosensory potentials, indicated a conduction block at the spinal level but produced inconclusive results. MRI revealed a posterior extra-axial spinal lesion extending from C6 to L1, appearing hypointense in T1 and hyperintense in T2. The lesion did not enhance with gadolinium but caused compression and displacement of the spinal cord towards the anterior region. Hyperintensity in T2 was observed, suggestive of myelitis from T4 to T11 (Figures 1-4).

**Figure 5:**
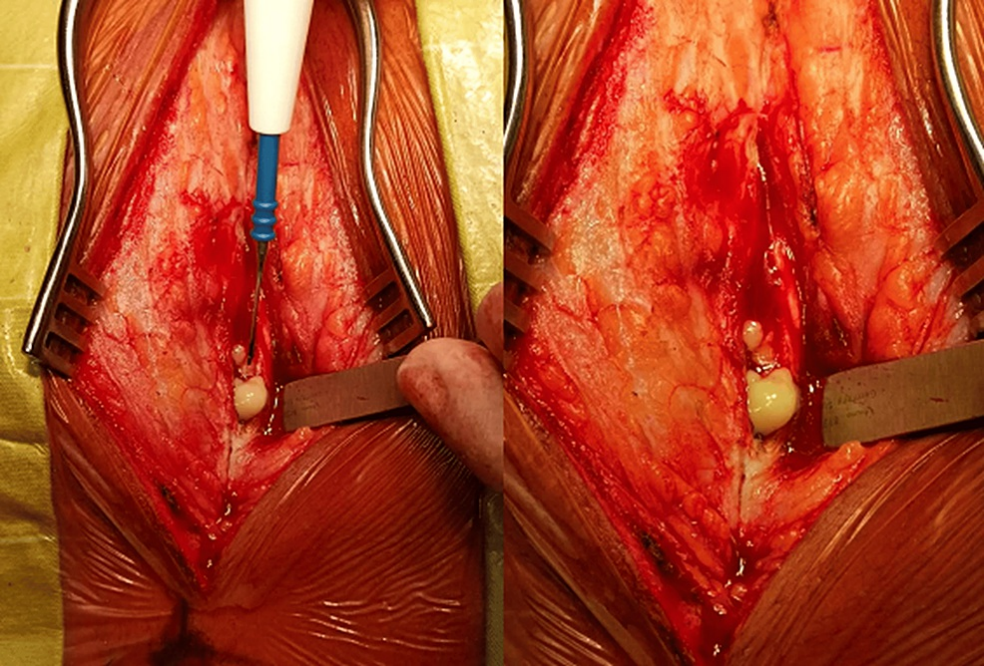
Transsurgical photo The image shows purulent material in the left paravertebral muscle at the T4-T5 level, with a fistulous tract entering the epidural space between the laminae of T4 and T5 through a spontaneous dural orifice

During the neurosurgical procedure, an abscess was observed in the left paravertebral muscle at the T4-T5 level, with a fistulous tract entering the epidural cavity between the laminae of T4 and T5 through a spontaneous dural hole (Figure 5). Purulent secretion was present in the intradural spinal canal with a thick, yellowish appearance. The surgical intervention involved resection of the spinous process and bilateral laminectomy of T5, bilateral hemilaminectomy of T6, and surgical cleaning with drainage of 12 cc of purulent fluid, which was sent for microbiological analysis. Culture and antibiogram of the purulent fluid from the spinal epidural space revealed methicillin-sensitive Staphylococcus aureus.

The postoperative course was characterized by a progressive decrease in leukocytes; however, the patient continued to have paraparesis and anesthesia in the limbs without functional improvement. On the third day, a suprapubic cystostomy was performed due to acute urinary retention. The patient was discharged and, in the following days, developed spastic paralysis in both lower limbs, remaining under outpatient surveillance with follow-up by the Neurosurgery and Infectious Disease services. Two months later, he was readmitted due to grade III sacral ulcers requiring surgical debridement, and no functional improvement in the lower limbs was reported.

## Discussion

SEA is an infection of the epidural space characterized by the accumulation of granulation tissue and/or pus between the dura mater and the vertebral periosteum [1]. The assessment of risk factors, including advanced age, lack of insurance, liver disease, alcoholism, HIV, and renal insufficiency, has shown high sensitivity and negative predictive value [5]. Diabetes mellitus, intravenous drug use, and local/systemic infections (e.g., respiratory, urinary, soft tissue) are the other significant sources of spinal epidural empyema. However, a substantial portion of cases has been found to have no identifiable cause of infection [2].

These abscesses occur within the epidural space between the dura mater and the vertebral periosteum, leading to direct mechanical compression or indirect ischemia of the spinal cord secondary to thrombophlebitis [6]. The most frequently identified pathogen is methicillin-sensitive Staphylococcus aureus (40%), both in blood cultures and surgical samples, followed by methicillin-resistant Staphylococcus aureus (30%) [5]. Bacteria enter the epidural space either through contiguous spread (in around one-third of the cases) or hematogenous dissemination (in approximately half of the cases), with the infection source remaining unidentified in the remaining cases. Abscesses are more likely to develop in larger epidural spaces containing fat, which is susceptible to infection [6].

A meta-analysis of thoracic SEA cases by Howie et al. reported that the most common symptoms are neurological deficits (68%), back pain (64%), and fever (24%); however, the triad of these findings is not specific to SEA [9]. Acute transverse myelitis is clinically characterized by symptoms and signs of acute or subacute development of neurological dysfunction in motor, sensory, and autonomic nerves, as well as spinal cord tracts [10]. Our patient presented over a week with motor and sensory dysfunction, paraplegia, anesthesia, and autonomic dysfunction characterized by constipation and acute urinary retention.

A well-established classification system outlines the progression of physical findings: in the initial stage, there is localized pain in the back at the level of the affected vertebral column; subsequently, there is radicular pain extending from the affected segment of the spinal cord, followed by impaired motor function, sensory loss, and dysfunction in bladder and bowel control. In the second stage, individuals with cervical or lumbar abscesses typically report neck pain radiating to the arms or lower back pain radiating to the legs. However, in the case of thoracic abscesses in the second stage, the presentation can be more elusive, manifesting as chest or abdominal pain [11], leading to diagnostic delays. The various stages of clinical presentation of SEA are outlined in Table 1.

**Table 1:** Stages of clinical presentation of spinal epidural abscess

Stages	Clinical manifestations
I	Non-specific pain and fever
II	Pain radiating to the segment of affected nerve roots
III	Motor deficit, sensory deficit, bladder, and bowel dysfunction
IV	Paralysis

In bacterial myelitis or spinal cord abscess, its origin has been postulated from a contiguous focus in the vertebral column, hematogenous spread, or secondary to bacteremia derived from a distant source. White blood cell count increases in only 13-60% of cases, that too moderately. While not crucial for diagnosis, white blood cell count can provide general guidance in assessing treatment response [12]. Bacteremia resulting from or originating from SEAs is identified in around 60% of patients, particularly in those with S. aureus infection compared to other organisms [13]. C-reactive protein (CRP) measurement can help differentiate serious causes of back pain, such as infections, cancer, and fractures, from mundane chronic back pain due to degenerative disease [14]. Measurement of CRP levels in blood has been found to help accelerate the diagnosis for patients with spinal column infections, including SEAs [15]. Lumbar puncture plays a less important role in diagnosing SEA and should not be routinely performed [4]. Typically, Gram staining of cerebrospinal fluid (CSF) yields negative results, and cultures of CSF are positive in less than 25% of patients undergoing microbiological evaluation of their CSF [6].

Gadolinium-enhanced MRI and myelography followed by CT of the spine exhibit high sensitivity (exceeding 90%) in detecting SEAs. Nevertheless, MRI is the preferred imaging modality due to its less invasive nature, ability to outline both the longitudinal and para-spinal extension of the abscess (crucial for surgical planning), and the capacity to differentiate between infection and cancer based on the appearance and intensity of the image signal [6]. In the case of SEA, two main patterns can be observed on MRI. One is the phlegmonous stage of the infection, which appears as a homogeneous enhancement of the affected area correlating with granulomatous tissue, microabscess, and pus accumulation. The other stage is the abscess surrounded by inflammatory tissue, showing a heterogeneous degree of peripheral enhancement with gadolinium. In this latter stage, the collection appears with a high T2 signal, with a generally low T1 signal surrounded by an enhancing rim. Diffusion-weighted imaging/apparent diffusion coefficient commonly demonstrates restricted diffusion of abscess content (Table 2) [16].

**Table 2:** Stages of spinal epidural abscess on MRI MRI: magnetic resonance imaging

Stages	MRI characteristics
Phlegmonous stage	T1 hypointense, T2 hyperintense, poorly defined lesion
Inflammatory stage	T1 isointense or hyperintense, T2 hyperintense, poorly defined lesion
Capsular stage	T1 hyperintense, T2 hyperintense, well-defined lesion

The treatment of choice is generally urgent surgical debridement combined with intravenous antibiotics [5]. Once the pathogen is identified, antibiotic therapy can be adjusted according to the susceptibility profile, and a switch to oral formulations (if sensitivity permits) can be considered after at least three weeks of intravenous administration. The usual duration of antibiotic treatment is 4-12 weeks [17]. In our case, targeted antimicrobial therapy was chosen based on the susceptibility profile. The preferred surgical procedure is laminectomy with debridement of infected tissues, as it represents a true neurosurgical emergency and should be performed as soon as possible [18]. It has been demonstrated in adults that there is a risk of deterioration with non-surgical management, even in patients for whom treatment is initiated in the absence of neurological deficits [19].

Medical management is only considered in neurologically intact patients for whom surgery is contraindicated due to comorbidities or in cases with high-risk surgery (infection with holocord distribution or anterior location of the abscess), or patients already neurologically compromised (>48 hours of paraplegia). If a conservative approach is chosen, monitoring of neurological status, inflammatory markers, and repeated MRI is mandatory [20].

## Conclusions

SEA is a rare condition, and hence a high index of clinical suspicion is required to detect it. Although various risk factors have been associated with this condition, it can manifest in patients without any of these factors, as described in our case. Medical personnel must identify early clinical signs, such as pain and motor or sensory deficits, and accurately interpret the results of blood tests and imaging studies to promptly initiate medical treatment with antibiotics and surgical intervention. The surgical approach involves posterior laminectomies at the affected spinal segments and abscess drainage. The goal is to prevent the persistence of neurological deficits in potentially salvageable patients with appropriate diagnostic and therapeutic interventions.
